# Expansion of phenotypically modified type 2 memory B cells after allergen immunotherapy

**DOI:** 10.1111/all.16320

**Published:** 2024-09-13

**Authors:** Anouk von Borstel, Simone Reinwald, Pei M. Aui, Craig I. McKenzie, Nirupama Varese, P. Mark Hogarth, Mark Hew, Robyn E. O'Hehir, Menno C. van Zelm

**Affiliations:** ^1^ Department of Immunology School of Translational Medicine, Monash University Melbourne Victoria Australia; ^2^ Allergy, Asthma and Clinical Immunology Alfred Health Melbourne Victoria Australia; ^3^ Immune Therapies Group Burnet Institute Melbourne Victoria Australia; ^4^ Department of Pathology The University of Melbourne Parkville Victoria Australia; ^5^ Department of Immunology Erasmus MC, University Medical Center Rotterdam the Netherlands

To the Editor,

Acute, type I hypersensitivity responses are mediated by immunoglobulin (Ig) E that binds to Fcε receptors (FcεRI and FcεRII) on immune effector cells.^1^ As IgE‐expressing memory B cells (Bmem) are very infrequent (0.01% of B cells) in human circulation,^2^ these are unlikely to be the sole reservoir of pathogenic immune memory.^3^ Two independent groups recently defined an IgG+ Bmem subset, expressing germline *IGHE* transcripts and surface CD23 (FcεRII) and IL‐4Rα.^4^, ^5^ These “type 2 Bmem” were expanded in allergic subjects. We previously observed an increase in allergen‐specific Bmem expressing CD23, IL‐4Rα and CD29 after successful allergen immunotherapy (AIT).^6^ Since AIT aims to redirect the B cell response by promoting IgG2 and/or IgG4 production instead of IgE,^7^ we here investigated allergen‐specific type 2 Bmem in bee venom (BVM) and ryegrass pollen (RGP) allergic patients, before and after commencing AIT.

We included 38 RGP‐allergic^6^, ^7^ and 17 BVM‐allergic patients^8^ with clinically evident allergic disease (Table S1) and confirmed allergen‐specific IgE (Figure 1A). Using recombinant Lol p 1 and Api m 1 protein tetramers,^9^ we conducted extensive flow cytometric immunophenotyping of total and allergen‐specific Bmem (Figure 1B and Figure S1A). Within allergen‐specific Bmem, a higher proportion of cells expressed CD23 than within total Bmem of both RGP‐ and BVM‐allergic patients (Figure 1C). This confirms the association of the type 2 phenotype with allergen specificity,^4^, ^5^ and expands the phenotype to new allergens and a new type of allergy (BVM).

**FIGURE 1:**
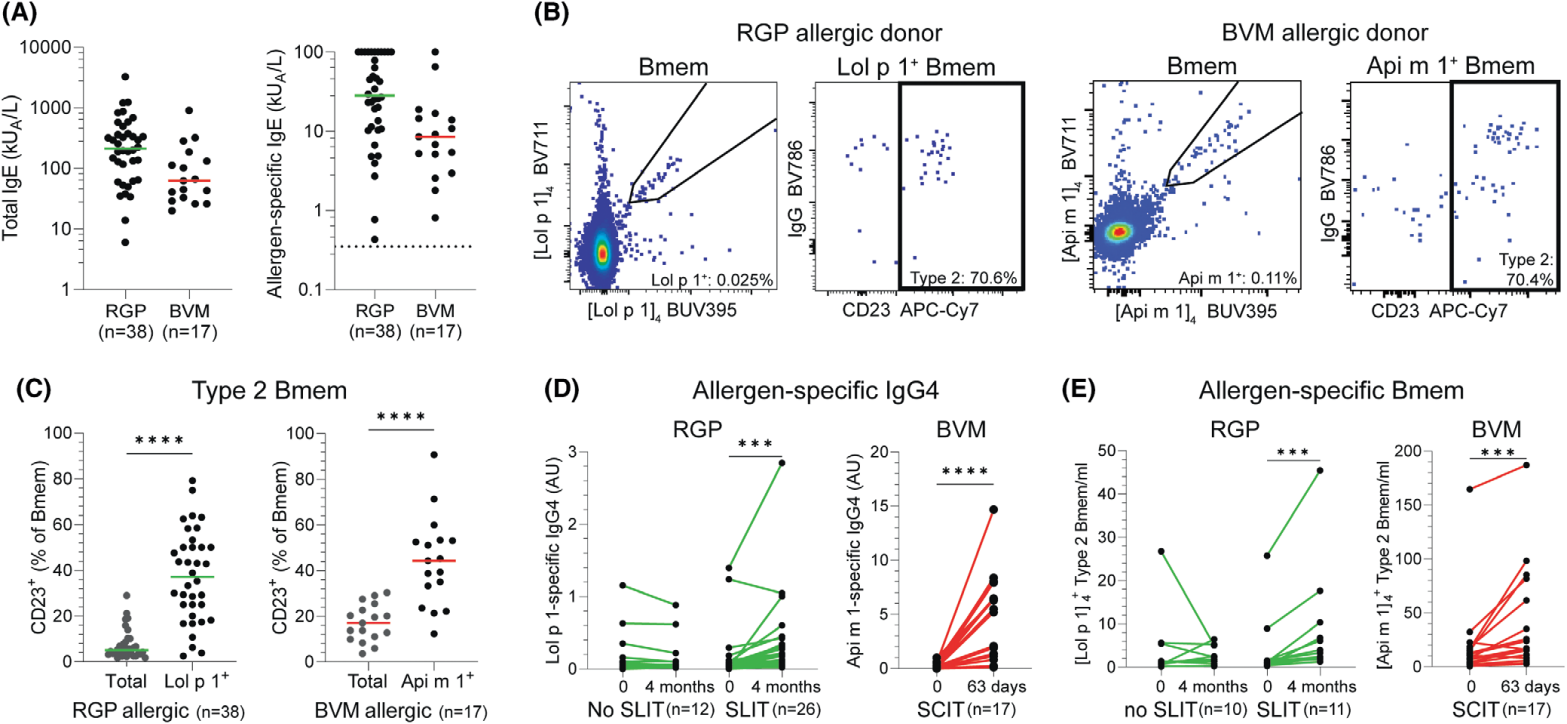
Allergen‐specific type 2 Bmem in allergy and after allergen‐immunotherapy. (A) Total IgE and allergen‐specific IgE (ImmunoCap) levels in kU_A_/L. (B) Double discrimination using fluorescent Lol p 1 tetramers (RGP) and Api m 1 tetramers (BVM) for identification of allergen‐specific Bmem, and CD23^+^ type 2 Bmem therein. (C) Frequencies of type 2 Bmem within total and Lol p 1^+^ Bmem (RGP) or Api m 1^+^ Bmem (BVM). (D) Serum Lol p 1‐specific IgG4 levels RGP‐allergic subjects before and after 4 months without SLIT (No SLIT) or with SLIT (left). Serum Api m 1 specific IgG4 levels for BVM‐allergic patients before and at 63 days on a SCIT regimen (right). (E) Absolute cell counts of allergen‐specific type 2 Bmem in RGP‐allergic patients who did not receive SLIT (No SLIT) and RGP‐allergic patients who received 4 months of SLIT, and in BVM‐allergic patients before and at 63 days on a SCIT regimen. Statistics: Wilcoxon matched‐pairs ranked test, ***p < .001, ****p < .0001.

We then examined the impact of AIT on type 2 Bmem, either on day 63 of ultra‐rush SCIT for BVM, or after 4 months of daily SLIT for RGP (*n* = 26). Twelve RGP‐allergic patients were assessed after 4 months pharmacotherapy only (i.e., No SLIT). RGP SLIT resulted in increased Lol p 1‐specific IgG4 serum levels (Figure 1D), as did BVM SCIT for Api m 1‐specific serum IgG4. Thus, both AIT regimens yielded the expected immunological changes.

The pharmacotherapy‐treated RGP‐allergic patient group did not show a change in total nor in Lol p 1^+^ type 2 Bmem numbers after 4 months (Figure 1E and Figure S1D). Following RGP SLIT, total (*p* < .05) and Lol p 1^+^ (*p* < .001) type 2 Bmem were increased, and after BVM SCIT only Api m 1^+^ type 2 Bmem were increased (*p* < .001).

As SLIT for RGP induces upregulation of CD29 and IgG4 on Lol p 1^+^ Bmem,^6^ we evaluated whether the expanded allergen‐specific type 2 Bmem populations were phenotypically altered (Figure 2A). A significantly higher percentage of allergen‐specific type 2 Bmem expressed CD29 after AIT for RGP (*p* < .05) or BVM (*p* < .01; Figure 2B), but not after pharmacotherapy for RGP allergy. Furthermore, IgG4^+^ frequencies within allergen‐specific type 2 Bmem increased following AIT for BVM and RGP, but not after RGP pharmacotherapy (Figure 2C). Total, not allergen‐specific type 2 Bmem were not phenotypically altered (Figure S1). Thus, AIT drives the expansion of allergen‐specific type 2 Bmem and induces a modification of the phenotype towards expression of CD29 and IgG4.

**FIGURE 2:**
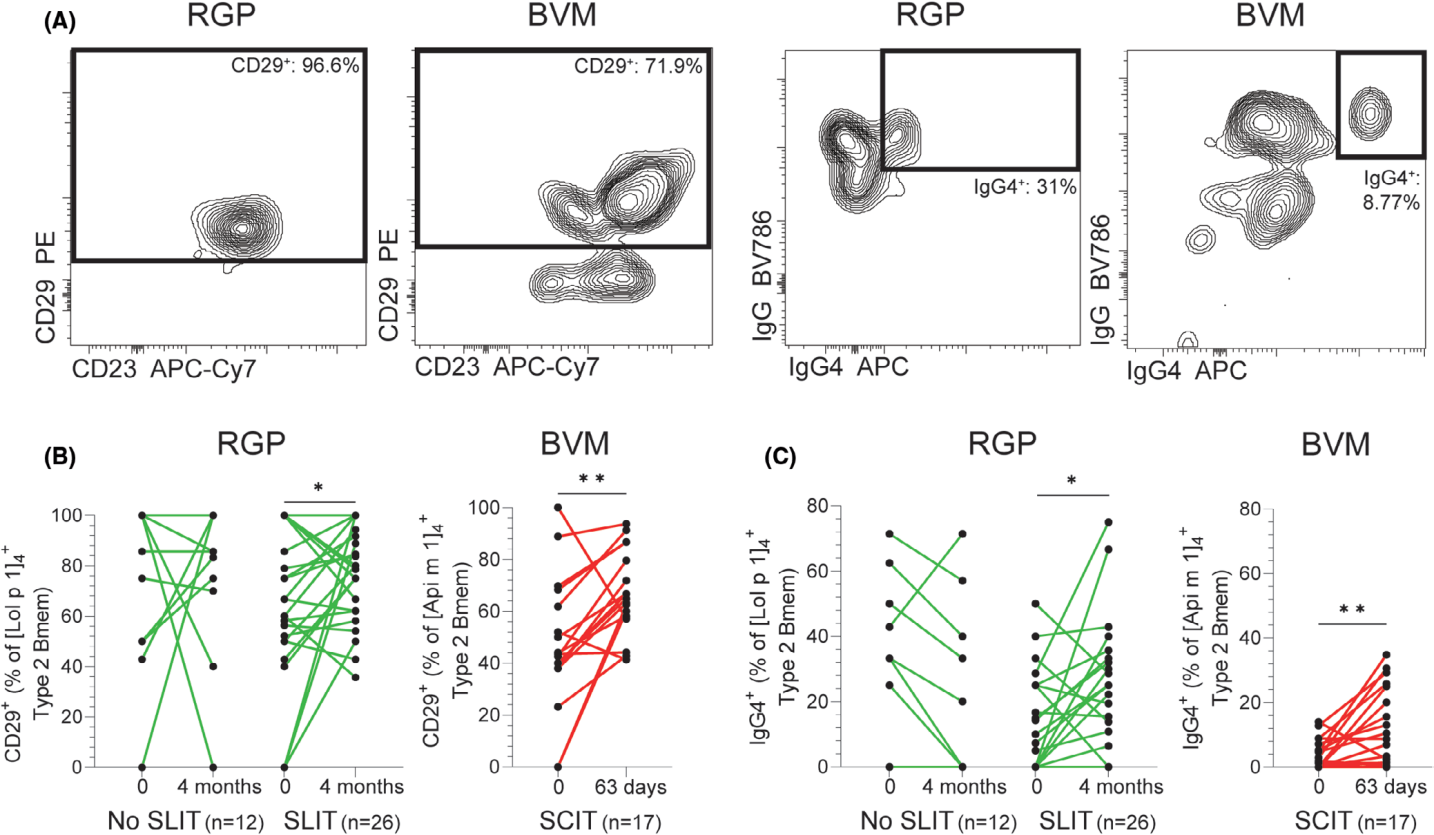
AIT modifies the phenotype of allergen‐specific type 2 Bmem. (A) Gating strategy of CD29^+^ and IgG4^+^ cells within allergen‐specific type 2 Bmem in RGP and BVM allergic donors. Frequencies of allergen‐specific type 2 Bmem expressing (B) CD29 or (C). IgG4. In green, RGP‐allergic patients who did not receive SLIT (No SLIT) and RGP‐allergic patients who received 4 months of SLIT. In red, BVM‐allergic patients before and at 63 days on a SCIT regimen. Statistics: Wilcoxon matched‐pairs ranked test, *p < .05, **p < .01.

Arguably, these phenotypic changes in type 2 Bmem after AIT contribute to the clinical effects.^10^ AIT is widely reported to induce allergen‐specific IgG4, and we here show for the first time that this is directly associated with the expansion of type 2 Bmem that express IgG4. Recently, CD29 was found to inhibit B cell activation, as mice with CD29‐deficicent B cells displayed enhanced B‐cell receptor (BCR) signaling upon stimulation ex vivo.^11^ Thus, upregulation of CD29 might inhibit activation of allergen‐specific type 2 Bmem.

Our evaluations after 4‐months of SLIT for RGP and 63 days of SCIT for BVM allergy are relatively early for regimens that are recommended for 3–5 years. While repeated antigen exposure seems to drive type 2 Bmem expansion, it remains unclear how these numbers are affected over a longer treatment period. Previously, CD23 expression on class‐switched Bmem in patients with allergic rhinitis was found to be reduced after 12 months of HDM‐SCIT and correlated with disease remission.^12^ Longitudinal studies of long‐term AIT (i.e., >1 year) in patients achieving a reduction in allergy symptoms are essential to elucidate the AIT effect on type 2 Bmem.

## AUTHOR CONTRIBUTIONS

MCvZ, AvB and REO'H conceived the idea for the present study. REO'H and MH recruited patients and facilitated sample collection. AvB, SR, PMA, CIM and NV analyzed the data. AvB and MCvZ wrote the manuscript with input from PMH, MH and REO'H. All authors revised and commented on manuscript drafts.

## FUNDING INFORMATION

This study was supported by an Early Career Postdoctoral Fellowship from the Faculty of Medicine, Nursing and Health Sciences, Monash University to AvB, and an NHMRC Ideas Grant (#2000773) to MCvZ, REO'H, and MH.

## CONFLICT OF INTEREST STATEMENT

MCvZ, CIM, and REO'H are inventors on a patent related to this work (PCT/AU2023/050439). All other authors declare no conflicts of interest.

## Supporting information


Data S1.

## Data Availability

The data that support the findings of this study are available from the corresponding author upon reasonable request.
