# Supplementary material for: Expansion of phenotypically modified type 2 memory B cells after allergen immunotherapy
Source: Allergy. 2024 Sep 13;80(3):867–9. doi: 10.1111/all.16320 (PMC11891399; doi:10.1111/all.16320)
Supplement: Supplementary file 1 — Data S1. [file ALL-80-867-s001.docx]

**SUPPLEMENTARY MATERIAL**

**METHODS**

**Study design**

The 38 patients with rye grass pollen (RGP) allergy were described previously.^1,2^ In short, all patients had serum RGP-specific immunoglobulin (Ig) E levels of >0.35 kU_A_/L (ImmunoCAP, Phadia), and moderate to severe allergic rhinitis (with or without asthma), and were recruited from the Allergy Clinics of The Alfred and Box Hill Hospitals (Alfred Ethics Committee project number 514/13). All patients were permitted standard pharmacotherapy with anti-histamines and topical intranasal corticosteroids and/or antihistamines for local symptom relief, and those patients who were in the treatment group (n=26) received sublingual immunotherapy (SLIT) as per standard protocol for 4 months prior to the pollen season. Peripheral blood samples were collected prior to the start of SLIT and after 4 months of treatment.

Seventeen patients with bee venom (BVM) allergy were recruited from the Allergy Clinic of The Alfred hospital (Alfred Ethics Committee project number 297/20). They were diagnosed on a positive history of a systemic hypersensitivity reaction to a bee sting and serum BVM-specific IgE level of >0.35 kU_A_/L (ImmunoCAP). All patients were treated with subcutaneous ultra-rush allergen immunotherapy (AIT) as per standard protocol. Peripheral blood samples were collected pre-AIT on day 0 and after 63 days of treatment.

The study was conducted according to the principles of the Declaration of Helsinki, and written informed consent from each participant was obtained prior to inclusion.

**Peripheral blood processing**

Heparinized blood samples were collected for each patient and processed within 24 hours. Absolute cell counts were determined for 10 No SLIT RGP-allergic, 11 SLIT-treated RGP-allergic, and 17 SCIT-treated BVM patients using the BD Trucount Absolute counting tubes (BD Biosciences, Franklin Lakes, NJ, USA) with 50 µl of heparinized blood and CD3, CD4, CD8, CD19, CD16, CD45, and CD56 antibodies.^3^ After following the manufacturer’s instructions, samples were measured on a LSRFortessa X-20 (BD Biosciences).

The remaining undiluted blood samples were centrifuged for 10 min at 1320 x g to separate plasma and cells. Plasma was collected and frozen at -80°C. Peripheral blood mononuclear cells (PBMC) were isolated by layering peripheral blood over Ficoll Paque (GE Healthcare, Chicago, IL, USA) and gradient centrifuged as per manufacturer’s instructions. The PBMC layer was washed twice in PBS before resuspension into 40% RPMI 1640, 50% fetal calf serum, and 10% DMSO and freezing in liquid LN_2_.

**Production of recombinant Lol p 1 and Api m 1**

Recombinant Lol p 1 and Api m 1 were produced in *Spodoptera frugiperda* 21 insect cells as described previously.^4^ In short, both proteins were generated with a 6-His tag for purification and a BirA tag for targeted biotinylation. Supernatant was collected and purified by application to a Talon NTA‐cobalt affinity column (Takara Bio, Kusatsu, Shiga, Japan) and subsequent elution with 200 mm Imidazole. Eluted protein was then dialyzed against 10 mm Tris for 48 h at 4°C, followed by biotinylation at room temperature overnight with 1/8 of final volume each of Biomix A (0.5 m Bicine‐HCl, pH 8.3) and Biomix B (100mM ATP, 100 mM MgOAc, 500 μm D‐biotin) and 2.5 μg of BirA enzyme per milligram of protein. Biotinylated protein was subsequently dialyzed against 10 mM Tris for 36 h at 4°C. Purified biotinylated Lol p 1 and Api m 1 proteins were tetramerized at a 4:1 molar ratio with fluorochrome-conjugated streptavidin.

**Plasma IgE level determination and ELISA**

Total and RGP- and BVM-specific IgE was determined in serum by ImmunoCAP for all patients as part of routine diagnostic patient care. Lol p 1-specific and Api m 1-specific IgG4 levels were determined by an in-house ELISA for 45 RGP-allergic patients (of which 28 receiving SLIT) and 18 BVM-allergic patients (the other patients had a missing serum sample at one timepoint) as described previously.^2^ In short, wells were coated with recombinant, monomeric Lol p 1 (MyBiosource, San Diego, CA, USA) or Api m 1, followed by blocking with 5% skim milk powder in PBS. Then, serially diluted plasma samples were incubated. Standard curves were generated with purified recombinant human IgG4 (clone AbD18705; Bio-Rad, Hercules, CA, USA) directly coated to the plate.^2^ Bound Lol p 1-specific antibodies were detected with biotinylated anti-hIgG4 (clone HP6025, Sigma-Aldrich, St. Louis, MO, USA) followed by addition of polyclonal goat anti-rabbit HRP (Promega, Madison, WI, USA). ELISAs were developed using TMB (Thermo Fisher Scientific) and the reaction was stopped with 1M HCl. Absorbance was measured at OD 450 nm (Multiskan Microplate Spectrophotometer; Thermo Fisher Scientific). The wells to which no allergens were added were used to determine background values, which were subtracted from allergen-specific immunoglobulin absorbance values.

**Flow cytometry**

Lol p 1- and Api m 1-specific memory B cells (Bmem) were immunophenotyped as described previously.^2^ Details of antibody reagents are provided in **Table S2**. In short, first CD19^+^ B cells were gated, followed by selection of CD38^dim^ mature B cells. Within this population, Bmem were defined by exclusion of IgD^+^CD27^-^ naive B cells (**Figure S1A**). Within the Bmem population, CD23^+^ cells were identified (**Figure S1A**) and within this subset, cells expressing CD29 or IgG4 (**Figure S1B-C**) were defined. In parallel, Lol p 1- or Api m 1 specific Bmem were defined by double discrimination,^5^ followed by evaluation of CD23, CD29 and IgG4. The RGP-allergic samples were acquired on a 5-laser LSR-Fortessa X-20 (BD Biosciences) and the BVM-allergic samples were acquired on a 5-laser Cytek Aurora spectral flow cytometer (Cytek Biosciences, Fremont, CA, USA). Using absolute B-cell counts per microliter blood as obtained by TruCount analysis (see above), B-cell subset frequencies were calculated into absolute cell counts. Data were analyzed with FlowJo Software (v10.8.1, BD Biosciences)

**Statistics**

All data were statistically analyzed using GraphPad Prism software (v 9.5.1, GraphPad Software, San Diego, CA, USA). As our data were not normally distributed (determined with Shapiro-Wilk test) and contained high variability, ranked tests were used to determine statistical significance: Mann-Whitney U test for unpaired data and a Wilcoxon matched-pairs ranked test for paired data. With such tests, outliers will not have a skewed effect on data analysis.

**Supplemental Tables** (n=2)

**Table S1. Characteristics of the RGP and BVM allergy patient cohorts.**

|  | RGP allergy | | BVM allergy |  |
| --- | --- | --- | --- | --- |
|  | No SLIT (n=12) | SLIT (n=26) | SCIT (n=17) |  |
| Median age (yr; range) | 25 (18 - 57) | 39 (18 - 65) | 58 (18 - 69) |  |
| Female | 83.33% | 58.69% | 29.41% |  |
| Seasonal AR | 100% | 100% | N/A |  |
| Asthma | 33.33% | 42.31% | N/A |  |
| Systemic hypersensitivity | N/A | N/A | 100% |  |
| Total serum IgE (kU_A_/L; median, range) | 276 (129-838) | 195 (6-3271) | 63 (26-906) |  |
| Allergen-specific IgE (kU_A_/L; median, range) | 45.8 (4.9-100) | 21.8 (0.4-100) | 8.5 (1.8-100) |  |
| AR, allergic rhinitis; BVM, bee venom; N/A, not applicable; RGP, ryegrass pollen; SCIT, subcutaneous immunotherapy; SLIT, sublingual immunotherapy | | | | |

**Table S2. Antibody panel for flow cytometry.**

| Target | Fluorochrome | Clone | Supplier |
| --- | --- | --- | --- |
| CD3 | BUV496 | UCHT1 | BD Biosciences |
| CD19 | BUV737 | SJ25C1 | BD Biosciences |
| CD23 | APC-Cy7 | EBVCS-5 | BioLegend |
| CD27 | BV421 | M-T271 | BD Biosciences |
| CD29 | PE | TS2/16 | BioLegend |
| CD38 | BV605 | HIT2 | BioLegend |
| IgD | PerCP-Cy5.5 | IA6-2 | BioLegend |
| IgG | BV786 | G18-145 | BD Biosciences |
| IgG4 | APC | S4G4 | Cytognos |
| Streptavidin | BUV395 | - | BD Biosciences |
| Streptavidin | BV711 | - | BD Biosciences |

**Supplemental Figure** (n=1)

**
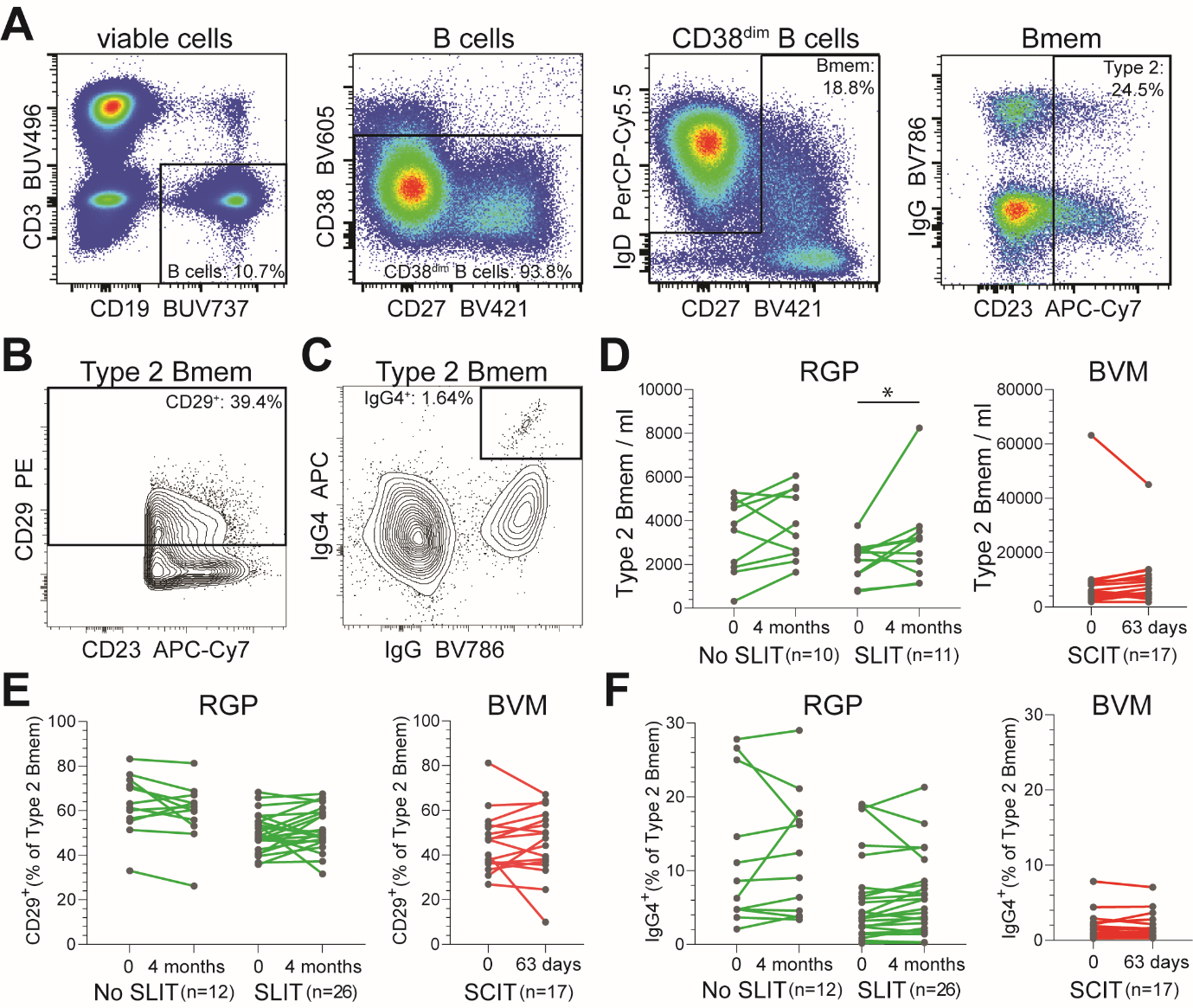
**

**Figure S1. Flow cytometric identification of total and allergen-specific Bmem subsets.** **A.** Detection of CD19^+^CD3^-^ B cells, followed selection of CD38^dim^ mature B cells. Within mature B cells, total memory B cells (Bmem) were defined by exclusion of IgD^+^CD27^-^ naive B cells. Within Bmem, the type 2 subset was defined as CD23^+^. Within total type 2 Bmem, cells were gated as **B.** CD29^+^ or **C.** IgG4^+^. **D.** The absolute cell counts (cells/mL) of total type 2 Bmem in No SLIT and SLIT RGP-allergic patient groups and BVM-allergic patients. **E.** CD29^+^ and **F.** IgG4^+^ frequencies within total type 2 Bmem. Statistics: Wilcoxon matched-pairs ranked test, *p<0.05

**Supplemental References**

1. Heeringa JJ, McKenzie CI, Varese N, et al. Induction of IgG(2) and IgG(4) B-cell memory following sublingual immunotherapy for ryegrass pollen allergy. *Allergy.* 2020;75(5):1121-1132.

2. McKenzie CI, Varese N, Aui PM, et al. RNA sequencing of single allergen-specific memory B cells after grass pollen immunotherapy: Two unique cell fates and CD29 as a biomarker for treatment effect. *Allergy.* 2023;78(3):822-835.

3. Edwards ESJ, Bosco JJ, Aui PM, et al. Predominantly Antibody-Deficient Patients With Non-infectious Complications Have Reduced Naive B, Treg, Th17, and Tfh17 Cells. *Front Immunol.* 2019;10:2593.

4. McKenzie CI, Varese N, Aui PM, et al. CytoBas: Precision component-resolved diagnostics for allergy using flow cytometric staining of basophils with recombinant allergen tetramers. *Allergy.* 2021;76(10):3028-3040.

5. van Zelm MC, McKenzie CI, Varese N, Rolland JM, O'Hehir RE. Advances in allergen-specific immune cell measurements for improved detection of allergic sensitization and immunotherapy responses. *Allergy.* 2021;76(11):3374-3382.
